# Can water-level management reduce malaria mosquito abundance around large dams in sub-Saharan Africa?

**DOI:** 10.1371/journal.pone.0196064

**Published:** 2018-04-19

**Authors:** Solomon Kibret, G. Glenn Wilson, Darren Ryder, Habte Tekie, Beyene Petros

**Affiliations:** 1 Ecosystem Management, University of New England, NSW, Armidale, Australia; 2 Department of Zoological Sciences, College of Natural Sciences, Addis Ababa University, Addis Ababa, Ethiopia; 3 Department of Microbial, Cellular and Molecular Biology, College of Natural Sciences, Addis Ababa University, Addis Ababa, Ethiopia; Bristol University/Remote Sensing Solutions Inc., UNITED STATES

## Abstract

**Background:**

Water level management has been suggested as a potential tool to reduce malaria around large reservoirs. However, no field-based test has been conducted to assess the effect of water level management on mosquito larval abundance in African settings. The objective of the present study is to evaluate the effects of water level drawdown rates on mosquito larval abundance.

**Methods:**

Twelve experimental dams were constructed on the foreshore of the Koka Dam in Ethiopia. These were grouped into four daily water drawdown treatments, each with three replicates: no water-level drawdown (Group 1; Control), 10 mm.d^-1^ (Group 2), 15 mm.d^-1^ (Group 3) and 20 mm.d^-1^ (Group 4). Larval sampling was conducted weekly for a period of 6 weeks each in the main malaria transmission season (October to November 2013) and subsequent dry season (February to March 2014). Larval densities were compared among treatments over time using repeated measures Analysis of Variance (ANOVA).

**Results:**

A total of 284 *Anopheles* mosquito larvae were collected from the experimental dams during the study period. Most (63.4%; n = 180) were collected during the main malaria transmission season while the remaining (36.6%; n = 104) were collected during the dry season. Larvae comprised four *Anopheles* species, dominated by *Anopheles arabiensis* (48.1% of total larval samples; n = 136) and *An*. *pharoensis* (33.2%; n = 94). Mean larval density was highest in control treatment dams with stable water levels throughout the study, and decreased significantly (*P* < 0.05) with increasing water drawdown rates in both seasons. During the main transmission season, anopheline larval density was generally lower by 30%, 70% and 84% in Groups 2, Group 3 and Group 4, respectively, compared with the control dams (Group 1). In the dry season, larval density was reduced by 45%, 70% and 84% in Groups 2, Group 3 and Group 4, respectively, when compared to the control dams.

**Conclusion:**

Increased water drawdown rates were associated with lower mosquito larval abundance. Water level management could thus serve as a potential control measure for malaria vectors around reservoirs by regulating the persistence of shallow shoreline breeding habitats. Dam operators and water resource managers should consider incorporating water level management as a malaria control mechanism into routine dam operations to manage the risk of malaria transmission to human populations around reservoirs.

## Introduction

Water storage can help safeguard livelihoods and reduce rural poverty [1]. Construction of dams has therefore been widely advocated to help ensure food security and promote economic development in Africa [2]. In recognition of this fact, the Program for Infrastructure Development in Africa (PIDA), endorsed in 2012 by the continent’s heads of state and government, has laid out an ambitious long-term plan for closing Africa’s infrastructure gap [3]. In the water and power sector, PIDA calls for an expansion of hydroelectric power generation capacity by more than 54,000 megawatts (MW) and that of water storage capacity by 20,000 cubic km. To meet these goals, Africa has entered a new era of dam building, with over 200 large dams currently under construction or planned for the near future [4]. The potential effect of large dams on malaria transmission, however, could potentially disrupt the intended benefits of these infrastructure projects in Africa [5–7].

A recent study quantified the impact of existing dams on malaria transmission in sub-Saharan Africa and found that dams contribute 1 million annual malaria cases each year in the region [6]. Dams particularly intensify malaria transmission in areas where the availability of mosquito breeding habitat is limiting [7] by providing ideal breeding sites for malaria vector species [8–10]. The long and shallow shorelines created by large impoundments often create areas of mosquito breeding habitat where it is difficult to control mosquito larvae using conventional methods. Such areas have been shown to attract the two most competent malaria vector mosquito species in Africa–*An*. *funestus* and *An*. *arabiensis* [7]. This is a serious concern in light of the region’s 2015–2030 malaria elimination aim to reduce malaria morbidity and mortality by 40% in 2020 and by 90% in 2030, compared with 2015 levels [11]. This highlights the need for tailor-made additional and complementary malaria control tools in order to mitigate malaria from major transmission foci associated with dams in the region.

Current malaria intervention strategies heavily rely on insecticide-treated bed nets and indoor residual insecticide spraying [12]. Whilst these vector control tools have contributed to the recent 60% decline in the global malaria mortality rates between 2001 and 2013, averting an estimated 6.1 million deaths [13], the increasing costs of these intervention tools has been challenging control efforts in high-burden developing countries. Furthermore, resistance of mosquitoes to available insecticides and parasites to drugs has often required the need for more expensive and new tools. With a tightening of global funds for malaria control in recent years [14], the need for supplementary cost-effective vector control measures has emerged as a priority.

In Africa, malaria transmission is unevenly distributed, often clustering around water bodies [15–17]. The association of malaria transmission with specific locations is attributable to the presence of breeding sites of the anopheline vectors, with water body characteristics playing an important role in determining the risk of malaria transmission. Households located nearest to larval sites are often at greater risk of the disease [18], as *Anopheles* mosquitoes have a limited dispersal distance–rarely exceeding 5 km in African settings [19]. Indeed, it is important to understand the site-specific characteristics relevant to malaria transmission. Recent research found that distance to reservoir shoreline is the most important environmental factor that explained 47% of the malaria incidence in villages within 5 km of the shoreline [20]. This highlights the need to explore reservoir management interventions that could feasibly reduce local rates of malaria transmission.

Utzinger et al. [21] identified that environmental management is the most cost-effective malaria control measure to substantially roll back malaria transmission in Africa. Strategies such as reservoir management through the optimization of dam operation regimes have been used in the Western world to mitigate malaria around large dams [22–24]. For example, the Tennessee Valley Authority in the United States effectively managed mosquito breeding associated with reservoirs by optimizing water level drawdown rates during the peak malaria transmission season [24]. However, there is no documented record of applying reservoir water level management for malaria control in African settings. With the current dam construction boom in Africa [4], it is timely to explore additional cost-effective tools such as reservoir management in order to supplement existing conventional malaria control measures.

The present study assessed water level management as a mechanism to reduce malaria vector abundance around reservoirs. Previous desktop-modeling predicted that water levels falling at a rate of 20 millimeters per day (mm.d^-1^) were associated with larval abundances approximately five-times lower than when water levels were reduced by 10 mm.d^-1^ [25]. However, these data were not tested in a field setting. Here, a field experiment was carried out to evaluate the effectiveness of four different rates of water drawdown on anopheline mosquito larval abundance in reservoir shorelines.

## Methods

This study was conducted around the Koka reservoir, about 100 km southeast of the capital Addis Ababa, in Central Ethiopia. Commissioned in 1960, it is a concrete gravity dam with a total capacity of 1850 million m^3^ and a mean annual regulated flow of 42.3 m^3^s^-1^. The dam was constructed primarily for hydropower generation with an installed capacity of 43.2 MW from three turbines (i.e., approximately 6% of the current total grid-based generating capacity of the country). The Wonji sugarcane irrigation scheme (6,000 ha), located approximately 12 km downstream of the dam, is also dependent on releases from it. In addition, the dam is also used for flood control.

Ejersa is a small rural town located on the western shore of the Koka reservoir. The area has a semi-arid climate, with an average daily temperature of 22°C and annual rainfall of 700–800 mm (Ethiopian Meteorology Agency, unpublished report). Malaria is the major public health challenge around Koka reservoir. *Anopheles arabiensis* and *An*. *pharoensis* are the most common malaria vector species, mainly breeding in shoreline puddles and rain pools [26]. *Plasmodium falciparum* is responsible for over 60% of malaria infections while the remaining malaria illnesses are due to *P*. *vivax* [25].

### Field experiment design

**Fig 1:**
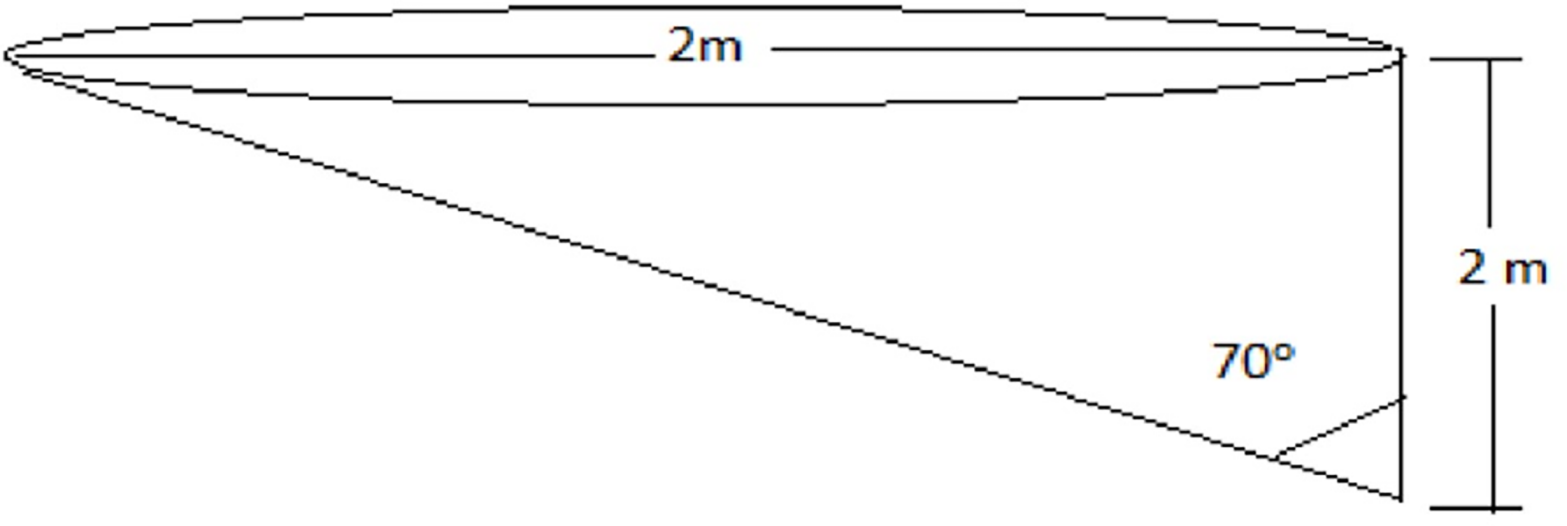
Schematic of the experimental dam.

**Fig 2:**
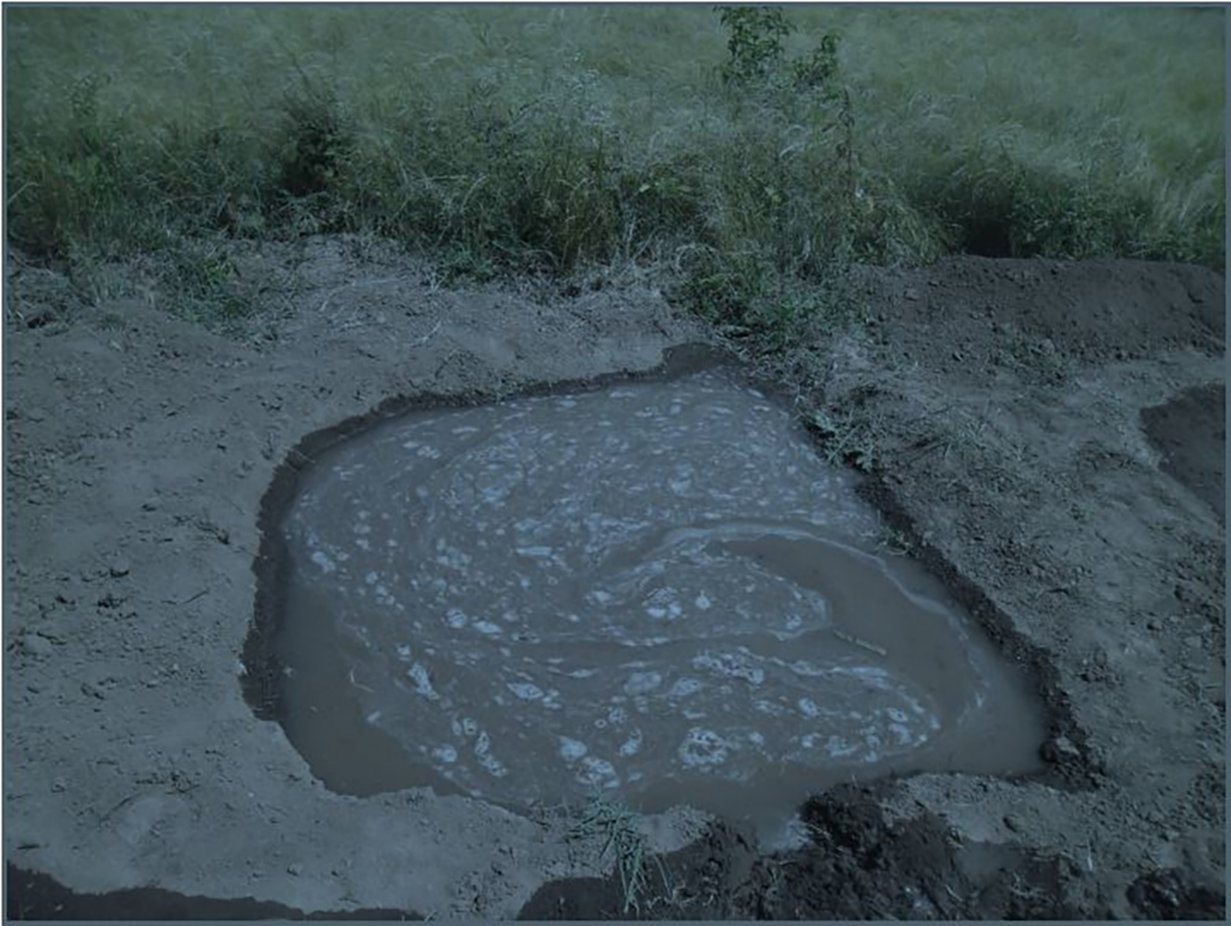
Experimental dam.

Twelve half-cone shaped experimental dams were built near the shoreline of Koka reservoir at Ejersa (Fig 1). These had a maximum depth of 2 m, a 1 m radius, and a bed slope of 70° to increase the shoreline benthic surface area for mosquito breeding (Fig 2). The experimental dams were dug 10 m apart from each other to ensure spatial independence, and filled with unfiltered water from the adjacent Koka shoreline. They were then left full for a month to allow conditions to stabilize before drawdowns and larval sampling was commenced.

The experiment was run for six weeks each in October to November 2013 (the main malaria transmission season) and February to March 2014 (the subsequent dry season). At the beginning of the first of these experimental periods, the 12 experimental dams were randomly allocated to one of four drawdown treatments (each with three replicates): no water-level drawdown (Control; Group 1), 10 mm.d^-1^ (Group 2), 15 mm.d^-1^ (Group 3) and 20 mm.d^-1^ (Group 4). These drawdown rates were adopted from a previous study on the Koka Dam that observed a significant reduction in mosquito larval abundance with a drawdown rate of 20 mm.d^-1^ during the main transmission season [25].

Drawdowns were effected by removing the required amount of water by bucket at approximately 7am each day throughout the two study periods. Volumes of water required to achieve a set drawdown rate were reduced each day for the duration of each study period due to the dams’ bed slope. Drawdown water was filtered and any mosquito larvae and other invertebrates returned to the experimental dam. Water levels in the control dams were kept constant by adding filtered water daily as required. Daily water depth was recorded from each experimental dam using a height gauge, to confirm each drawdown rate and allow calculation of inundated benthic surface area.

### Larval mosquito sampling

Weekly larval mosquito sampling was undertaken using a 350 mL standard dipper [27]. At each sampling, a total of six independent dips were taken around the edge of each of the experimental dams between 11:00 and 12:00 hours. Larvae were counted and all non-anophelines were discarded. Anopheline larvae in each sample were preserved in 70% alcohol for later species identification. In the laboratory, anopheline larvae were sorted to species based on morphological characteristics [28] and counted.

### Statistical analysis

Anopheline larval density was expressed as the number of larvae per m^2^ of experimental dam wetted surface area. Log-transformed larval density was compared among groups as well as seasons using repeated measures Analysis of Variance (ANOVA), followed by post hoc Tukey's Honestly Significant Difference (HSD) tests. The same analyses were used to compare differences between weekly water drawdown rate and larval density for the two major malaria vector species (*An*. *arabiensis and An*. *pharoensis*). To determine changes in larval density relative to water drawdown rate, Odds Ratio (OR) was calculated for each treatment group by using the control group as reference. All analyses were carried out using SPSS statistical software version 22 (SPSS Inc, Chicago, IL, USA) and Microsoft Excel 2010.

### Ethics approval and consent to participate

Not applicable

## Results

**Fig 3:**
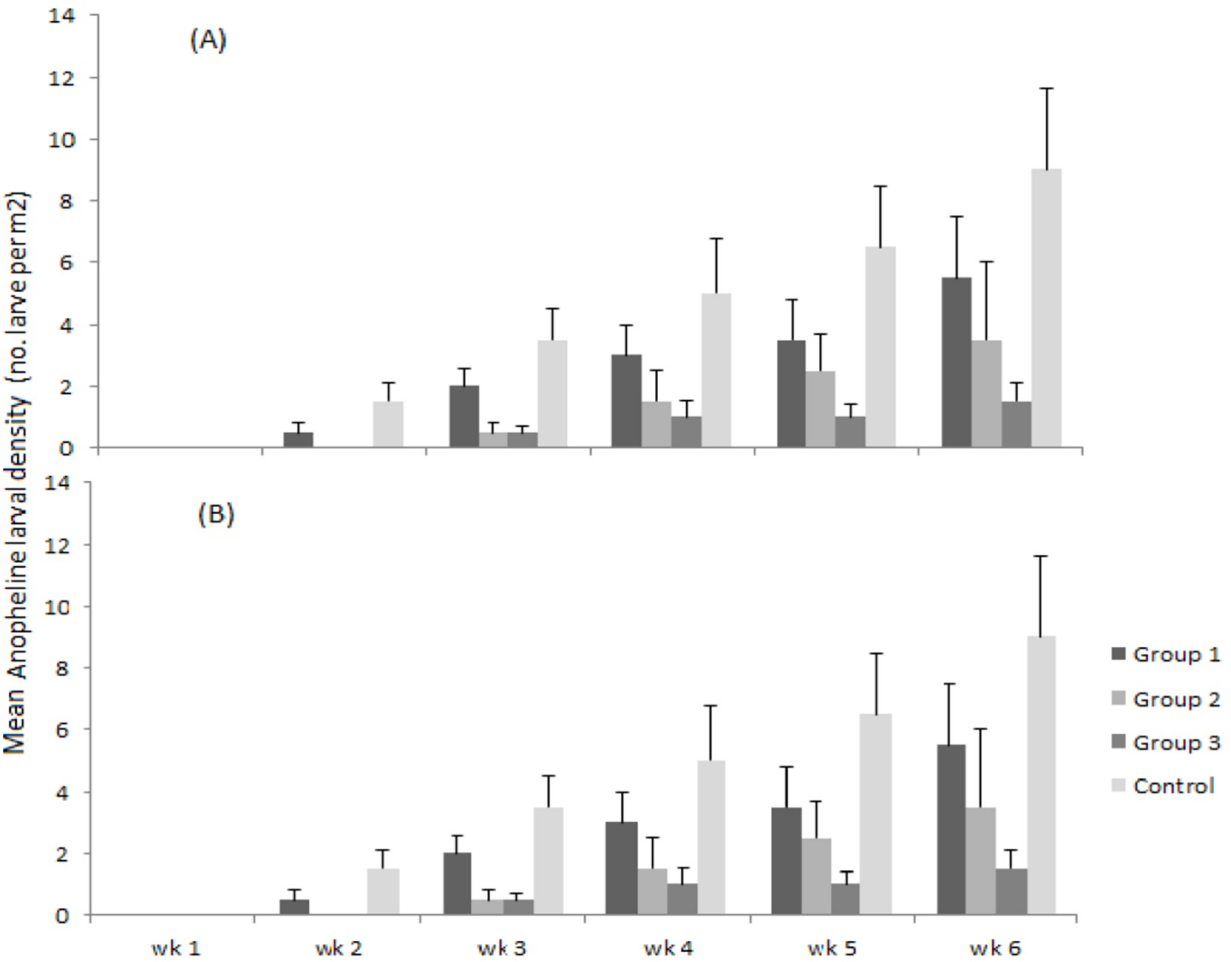
Mean anopheline larval density in the four groups of experimental dams with different water drawdown rates during the dry and main transmission season: (A) main transmission season (October-November 2013); (B) dry season (February-March 2014). Vertical bars indicate standard error.

A total of 284 anopheline larvae were collected from the experimental dams during the study (Table 1). Of these, 63.4% (n = 180) were collected during the main malaria transmission season while the remaining 36.6% (n = 104) were collected during the dry season. Among the four treatment groups, anopheline larval density was the highest in Group 1 (repeated measures ANOVA, degree of freedom (df) = 3; F = 6.58; *P* < 0.001), and declined significantly with increasing drawdown rate in Groups 2 to 4 (Tukey's HSD test, *P* < 0.001). A higher larval density in all experimental dam groups was found during the main transmission season (ANOVA; df = 1; F = 11.34; *P* < 0.01) compared with the dry season (Fig 3). Larval anophelines appeared in the second week and increased throughout sampling weeks in both seasons.

**Table 1:** Summary of weekly anopheline larval sampling from 12 experimental dams in Koka area, Central Ethiopia, during the main malaria transmission (October-November 2013) and dry (February-March 2014) seasons.

Water drawdown rate	Group 1 (control)	Group 2 (10 mm.day^-1^)	Group 3 (15 mm.d^-1^)	Group 4 (20 mm.d^-1^)	Total (%)
Main malaria transmission season (%)	78 (43)	56 (31)	28 (16)	18 (10)	180 (63)
Dry season (%)	51 (49)	29 (28)	16 (15)	8 (8)	104 (37)
Total (%)	129 (45)	85 (30)	44 (15)	26 (9)	284 (100)

During the main malaria transmission season, 43% (n = 78) of all anopheline larvae were collected from control dams, while 31% (n = 56), 16% (n = 28) and 10% (n = 18) were collected from experimental dams with 10 mm.day^-1^, 15 mm.day^-1^ and 20 mm.day^-1^ water drawdown rates, respectively. Similarly, almost half (49%; n = 51) of the anopheline larvae collected during the dry season were from control dams while 28% (n = 29), 15% (n = 16) and 8% (n = 8) were collected from the experimental dams in Group 2, 3 and 4, respectively.

Compared with the control (Group 1), larval density was generally reduced by 30% (OR = 0.70; *P* < 0.05), 70% (OR = 0.30; *P* < 0.05) and 84% (OR = 0.16; *P* < 0.05) in Groups 2, 3 and 4, respectively, during the main transmission season (Table 2). Similarly in the dry season, larval density was reduced by 45%, 70% and 84% in Groups 2, Group 3 and Group 4 when compared with the control (Group 1). Overall, larval abundance in control dams was 1.4–1.8 times higher than Group 2, 3.3 times higher than Group 3 and 6.1 times higher than Group 4.

**Table 2:** Comparison of mean larval density between treatment and control groups with different water drawdown rate in different seasons. [Group 1 (control) = 0 mm.d^-1^; Group 2 = 10 mm.d^-1^; Group 3 = 20 mm.d^-1^; Group 4 = 20 mm.d^-1^. SE refers to standard error].

	Total no. larvae	Mean larval density (±SE)	Odds Ratio	*P*
**Main transmission season**				
Group 1	78	6.1 (±2.0)	1	-
Group 2	56	4.3 (±1.7)	0.70	<0.05
Group 3	28	1.8 (±0.5)	0.29	<0.05
Group 4	18	1.0 (±0.3)	0.16	<0.05
**Dry season**				
Group 1	51	4.3 (±1.1)	1	-
Group 2	29	2.4 (±0.6)	0.56	<0.05
Group 3	16	1.3 (±0.4)	0.30	<0.05
Group 4	8	0.7 (±0.2)	0.16	<0.05

**Fig 4:**
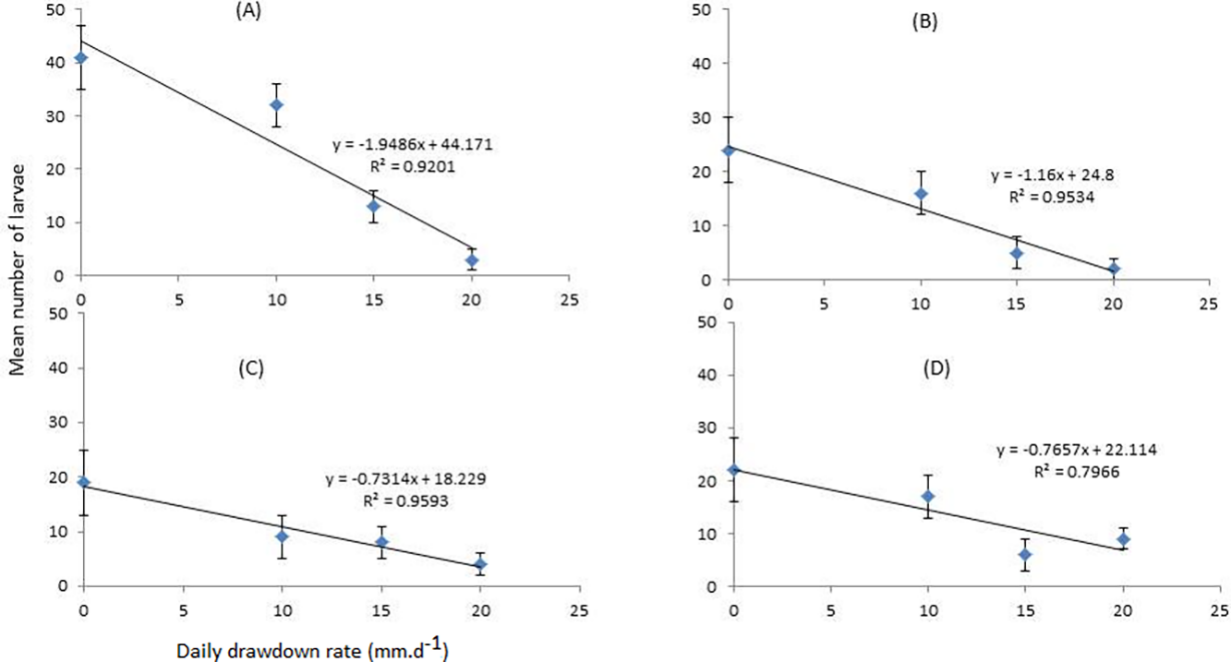
*Anopheles* vector larval abundance in experimental dams with different water drawdown rates. (A) *An*. *arabiensis* during the main transmission season; (B) *An*. *arabiensis* during the dry season; (C) *An*. *pharoensis* during the main transmission season; (D) *An*. *pharoensis* during the dry season.

Four *Anopheles* species were found breeding in the experimental dams during the study period (Table 3). *Anopheles arabiensis* was the dominant species, constituting 48.1% (n = 136) of the total larval samples, followed by *An*. *pharoensis* (33.2%; n = 94), *An*. *coustani sensu lato* (17.3%; n = 49) and *An*. *funestus sensu lato* (1.4%; n = 4). Both *An*. *arabiensis* and *An*. *pharoensis* were more abundant in control experimental dams compared to other experimental treatments (Fig 4). The differences in the occurrence of *An*. *arabiensis* (repeated measures ANOVA; df = 3; F = 14.76; *P*< 0.05) and *An*. *pharoensis* (df = 3; F = 9.49; *P* < 0.05) were significant across the four groups of experimental dams: larval abundance increased with slower water drawdown rates. This trend was significant (ANOVA; *P* < 0.01) in both the dry and main transmission seasons for both of the main two vector species.

**Table 3:** *Anopheles* species occurrence in experimental dams with different water level drawdown rates in different seasons.

	Main malaria transmission season	Dry season	Total
**Group 1 (0 mm.d**^**-1**^**)**			
*An*. *arabiensis*	41	24	65
*An*. *pharoensis*	22	19	41
*An*. *funestus*	3	0	3
*An*. *coustani*	12	7	19
Total	78	51	129
**Group 2 (10 mm.d**^**-1**^**)**			
*An*. *arabiensis*	32	16	48
*An*. *pharoensis*	17	9	26
*An*. *funestus*	0	0	0
*An*. *coustani*	7	4	11
Total	56	29	85
**Group 3 (15 mm.d**^**-1**^**)**			
*An*. *arabiensis*	13	5	18
*An*. *pharoensis*	6	8	14
*An*. *funestus*	1	0	1
*An*. *coustani*	8	3	11
Total	28	16	44
**Group 4 (20 mm.d**^**-1**^**)**			
*An*. *arabiensis*	3	2	5
*An*. *pharoensis*	9	4	13
*An*. *funestus*	0	0	0
*An*. *coustani*	6	2	8
Total	18	8	26

## Discussion

The present study found that faster water drawdown rates were consistently associated with reduced anopheline larval abundances. Water drawdown rates of 10 mm.d^-1^, 15 mm.d^-1^ and 20 mm.d^-1^ were associated with 30–45%, 70% and 84% reductions in larval density, respectively, when compared to control group–confirming that increasing drawdown rates results in a significant reduction in larval density. Larval abundance of both *An*. *arabiensis* and *An*. *pharoensis*, the two important malaria vector species in Ethiopia, also decreased as water drawdown rate increased.

Findings of the present study are consistent with those from previous research around Koka Dam [25] that suggested larval abundances would decline with higher rates of water drawdown, while increasing and stable water levels were associated with mounting larval densities. Kibret *et al*. [25] found that water levels falling at a rate of 10 mm.d^-1^ and 20 mm.d^-1^, respectively, are associated with larval abundances approximately 5 and 2.5 higher than when water levels fall at 25 mm.d^-1^ [25]. Similarly, a recent laboratory-based study indicated that water drawdown rates affect mosquito breeding [29]. The present study confirmed the potential efficacy of water level management as a control mechanism for malaria mosquito breeding in African settings.

Results of this study also indicated that larval densities of malaria vector mosquitoes (*An*. *arabiensis* and *An*. *pharoensis*) were significantly lower under faster rates of water drawdown. This is primarily because these mosquito species prefer still and stagnant breeding habitats, and so are susceptible to stranding and desiccation when water levels recede [30]. A slowly receding shoreline, as a consequence of slow water level drawdown rates, creates a prolonged wetted area and fringe of transient puddles where mosquito larvae could develop into the adult stage. In contrast, a rapidly receding shoreline would be expected to result in a desiccated shoreline less favorable for mosquito recruitment. In a laboratory controlled study, Endo *et al*. [29] noted that larger proportions of *Anopheles* larvae were stranded at higher water drawdown rates. Further study is required to assess the implication of water level management on dams and its impact on a reservoir’s primary purposes. Reis *et al*. [31] investigated the water resources implications of reservoir water level management through hydrological control to reduce malaria around the Koka Dam and found that the targeted use of hydrological control for malaria vector management would have negligible impacts on power generation and downstream irrigation industries. Nevertheless, the actual impact of reservoir management has not been determined for large dams in African settings.

With over 1 million malaria cases already associated with dams annually [6] and the recent release of the Global Malaria Action Plan for 2016–2030 by the Roll Back Malaria Partnership seeking to shrink malaria occurrence and ultimately eliminate the disease in Africa [11], the role of large dams in malaria transmission should not be overlooked. Recent data indicated that Africa is planning to build about 200 more large dams in next 5–10 years [4], taking the total to over 2000 dams by 2025. Tailor-made malaria control approaches are thus needed to curb the impact of planned and existing dams in Africa as the current conventional control tools are resource-intensive. The need to supplement the existing conventional malaria intervention tools (i.e. bednets and indoor residual spraying) has been particularly underscored because of the fact that these tools only target indoor-biting and/or resting mosquito species [12,32]. Larval management could thus further suppress malaria transmission by targeting the aquatic stages through reducing larval habitats, leading to a reduction of both outdoor- and indoor-biting vectors [33,34].

Larval source reduction using reservoir management is also cost-effective and is not based on a large workforce or intensive resources required by other conventional malaria control tools. Keiser *et al*. [33] reviewed the literature to evaluate the efficacy of larval management (i.e. methods creating temporary unfavorable conditions for mosquito breeding–e.g. water or vegetation management) in reducing malaria morbidity and mortality, and found that the risk of malaria was reduced by 88% in areas that actively applied environmental management tools for malaria control. The World Health Organization has been encouraging countries to use integrated vector control in their rational decision-making process for the optimal use of resources for vector control [12].

Although this study was repeated over two seasons, the total sample size of mosquito larvae collected in this experimental work was low. Thus, caution should be taken in extrapolating our findings, and we recommend that the findings of this study be confirmed in larger-scale studies with a larger sample size of mosquitoes. The present study was also limited in that it did not take into account other factors that can influence mosquito larval abundances around a reservoir shoreline, including wind conditions, wave action, shoreline slope, the occurrence of mosquito larvae predators and soil type [35]. For instance, a recent study around Koka Dam indicated that wind is an important factor underpinning larval development success along the shoreline [36]. Strong winds create waves that disrupt the maintenance of shoreline habitat for aquatic-stage mosquitoes. Similarly, shorelines with a sleep slope are less suited to larval survival as their poor water-holding capacity means they do not remain moist for long enough for the aquatic-stages to complete their development. The experimental dams constructed in this study may not have been subject to all (or any) of these effects, and the present findings need to be viewed with this caveat in mind. Equally, vegetation cover was one of the most important environmental factors underpinning the presence of *An*. *arabiensis* in Kenya [37]. The experimental dams were constructed along a relatively open portion of the reservoir shoreline, and the extent to which ground vegetation and nearby scattered larger plants was not considered.

Future studies should include an assessment of the effectiveness of water level management for larval control at different eco-epidemiological settings. Importantly, however, any testing of the present findings should be undertaken at the scale of whole reservoirs as the experimental dams used here are a simplistic representation of ‘real World’ conditions. Reservoir operations are based on rule curves and standard operating procedures, whereby the goal is to reach a certain release and storage targets at the end of a month (or season) given the antecedent conditions [2]. These targets are based on multi-stakeholder agreement and objective optimization. Each stakeholder (e.g. farming, water supply, hydropower, flood control) will have their own targets, often competing with each other (hydropower and flood control are good examples). In this sense, mosquito control objectives will represent a further stakeholder to a reservoir’s water use, and how water level fluctuation for mosquito control purposes might affect a reservoir’s primary purpose will need to be ascertained. At the Koka Dam, Reis *et al* [31] indicated that hydropower generation would increase as more water was released through the turbines because losses from spill, seepage, and evaporation were reduced as a consequence of more rapid drawdown of the reservoir at the end of the wet season. Yet, further study is required to assess how best to manage the conflicting objectives of water conservation and mosquito control. The massive shoreline wetland created by dams is likely to reduce the efficacy of other malaria vector control measures such as larviciding for routine application. The overall impact of incorporating malaria control into routine dam operation regimes, thus, needs to be examined.

In conclusion, water level management has potential as a malaria mosquito control tool if applied to large reservoirs. Faster water drawdown rates are associated with lower mosquito larval abundance, which ultimately reduces the malaria risk to human populations surrounding reservoirs. Dam operators and decision makers should consider incorporating malaria control mechanisms into routine dam operation practice without sacrificing the primary purpose of the water reservoir.

## Abbreviations

ANOVA: Analysis of variance
HSD: Honestly Significant Difference
MW: Megawatt
mm.d^-1^: millimeter per day
PIDA: Program for Infrastructure Development in Africa
